# Microbial Organic Matter Degradation Potential in Baltic Sea Sediments Is Influenced by Depositional Conditions and *In Situ* Geochemistry

**DOI:** 10.1128/AEM.02164-18

**Published:** 2019-02-06

**Authors:** Laura A. Zinke, Clemens Glombitza, Jordan T. Bird, Hans Røy, Bo Barker Jørgensen, Karen G. Lloyd, Jan P. Amend, Brandi Kiel Reese

**Affiliations:** a Department of Life Sciences, Texas A&M University Corpus Christi, Corpus Christi, Texas, USA; b Department of Biological Sciences, University of Southern California, Los Angeles, California, USA; c Center for Geomicrobiology, Department of Bioscience, Aarhus University, Aarhus, Denmark; d Department of Microbiology, University of Tennessee Knoxville, Knoxville, Tennessee, USA; e Department of Earth Sciences, University of Southern California, Los Angeles, California, USA; Shanghai Jiao Tong University

**Keywords:** Baltic Sea, heterotrophy, microbial ecology, sediment

## Abstract

Globally, marine sediments are a vast repository of organic matter, which is degraded through various microbial pathways, including polymer hydrolysis and monomer fermentation. The sources, abundances, and quality (i.e., labile or recalcitrant) of the organic matter and the composition of the microbial assemblages vary between sediments. Here, we examine new and previously published sediment metagenomes from the Baltic Sea and the nearby Kattegat region to determine connections between geochemistry and the community potential to degrade organic carbon. Diverse organic matter hydrolysis encoding genes were present in sediments between 0.25 and 67 meters below seafloor and were in higher relative abundances in those sediments that contained more organic matter. New analysis of previously published metatranscriptomes demonstrated that many of these genes were transcribed in two organic-rich Holocene sediments. Some of the variation in deduced pathways in the metagenomes correlated with carbon content and depositional conditions. Fermentation-related genes were found in all samples and encoded multiple fermentation pathways. Notably, genes involved in alcohol metabolism were amongst the most abundant of these genes, indicating that this is an important but underappreciated aspect of sediment carbon cycling. This study is a step towards a more complete understanding of microbial food webs and the impacts of depositional facies on present sedimentary microbial communities.

**IMPORTANCE** Sediments sequester organic matter over geologic time scales and impact global climate regulation. Microbial communities in marine sediments drive organic matter degradation, but the factors controlling their assemblages and activities, which in turn impact their role in organic matter degradation, are not well understood. Hence, determining the role of microbial communities in carbon cycling in various sediment types is necessary for predicting future sediment carbon cycling. We examined microbial communities in Baltic Sea sediments, which were deposited across various climatic and geographical regimes to determine the relationship between microbial potential for breakdown of organic matter and abiotic factors, including geochemistry and sediment lithology. The findings from this study will contribute to our understanding of carbon cycling in the deep biosphere and how microbial communities live in deeply buried environments.

## Abstract (precis)

Sediments sequester organic matter over geologic time scales and impact global climate regulation. Microbial communities in marine sediments drive organic matter degradation, but the factors controlling their assemblages and activities, which in turn impact their role in organic matter degradation, are not well understood. Hence, determining the role of microbial communities in carbon cycling in various sediment types is necessary for predicting future sediment carbon cycling. We examined microbial communities in Baltic Sea sediments, which were deposited across various climatic and geographical regimes to determine the relationship between microbial potential for breakdown of organic matter and abiotic factors, including geochemistry and sediment lithology. The findings from this study will contribute to our understanding of carbon cycling in the deep biosphere and how microbial communities live in deeply buried environments.

## INTRODUCTION

Organic matter (OM) burial in marine sediments sequesters carbon over geologic time and thereby plays a role in climate regulation. Globally, marine sediments store 7.8 × 10^22^ g of carbon, including organic matter from both terrestrial and marine sources (1). Marine OM is generally more nitrogen rich than terrestrial OM. It contains carbohydrates and proteins derived largely from water column organisms, compared with carbohydrates, such as cellulose and lignin, which are derived from vascular plants in the terrestrial component (1, 2). The contributions of these distinct organic pools to marine sediment varies between locations, climates, and geologic times (see, e.g., references 2–5). How these sources impact sedimentary carbon cycling and resident organisms is an area of active research.

The marine sedimentary biosphere holds an estimated 5 × 10^29^ prokaryotic cells (6, 7). Their metabolisms vary between sediments, depending partially on nutrient, electron acceptor, and electron donor availabilities (8–10). Surface microbial communities, temperature, recalcitrance of sediment OM, and depositional conditions also influence the composition and activities of the sedimentary biosphere (11–15). In near-shore environments, such as inland seas and along continental margins, organic loading to the sediment drives the development of the microbial “anaerobic food web” (see references 16 and 17 and references therein). In organic-rich sediments, microbes in the upper few meters of sediment below the seafloor use electron acceptors [e.g., O_2_, NO_3_^−^, Mn(IV), Fe(III), and SO_4_^2−^] in order of declining energy yields from organic matter respiration, ending with methanogenesis as the dominant process (18). Throughout the sediment column, heterotrophic metabolisms are critical to breaking down the complex macromolecules and producing smaller organic compounds, which feed into respiration and methanogenesis (19). However, it is difficult to distinguish between heterotrophic pathways *in situ* due to the large range of bioavailable organic substrates, the micromolar concentrations of substrates and products, the diversity of active microbial populations, and the number of pathways involved in OM remineralization in sediments (20–23). Recently, advances in molecular biology (e.g., metagenomics and metatranscriptomics), enzymatic assays, and organic geochemistry and analytical chemistry have allowed for more detailed studies of environmental OM degradation by microorganisms (24–26).

**FIG 1:**
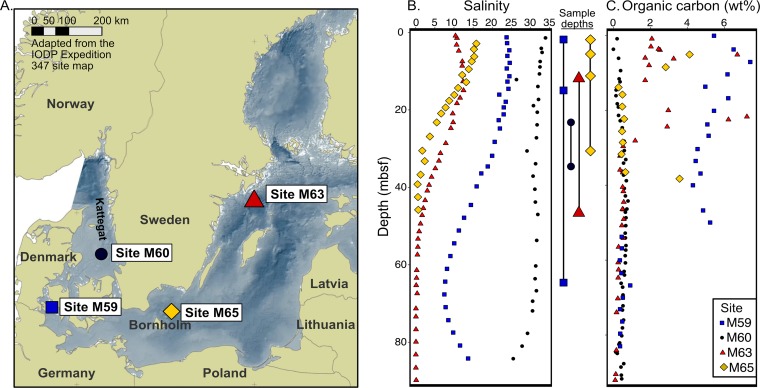
(A) Map of the samples locations in the Kattegat and Baltic Sea Basin (map adapted from the IODP Expedition 247 site map [2] [coastline: ESRI data and maps, 2005; bathymetry: BALANCE project, www.helcom.fi]). (B and C) Chlorinity-based pore water salinity (B) and total organic carbon (TOC) content (C) in percent dry weight and of sediments from IODP Expedition 347. Sample depths are indicated in the space between panels B and C and correspond to the key in panel C. Values for salinity and TOC were collected and reported as part of IODP Expedition 347 (38).

The Baltic Sea is an ideal location to study microbial organic remineralization due to its distinct and well-defined geological history as well as varied OM concentrations (27) (Fig. 1C). The Baltic Sea is a shallow intracontinental sea which receives terrestrial inputs from rivers and runoff and marine inputs from the North Sea via the Skagerrak and Kattegat bodies of water (28). This has created a salinity gradient both laterally into the Baltic Sea and vertically in the water column (29). Regional anoxia is frequent in the deepest basins, and the sediments are rich in OM due to eutrophication and high sedimentation rates (up to 500 cm per 1,000 years) (30). Towards the end of the last glaciation, the melting of the Scandinavian Ice Sheet caused dramatic environmental changes in the Baltic region. Approximately 16,000 years ago, as the basin was still partially covered by the Scandinavian Ice Sheet, the glacial Baltic Ice Lake started to form (31). Between the start of the Holocene 11,700 years before present (BP) to approximately 10,700 BP, a connection of the ice lake to the North Sea caused a brief brackish phase of the basin, the Yoldia Sea (32). This was followed by the low-primary-productivity freshwater Ancylus Lake phase. Sediments deposited in both the Baltic Ice Lake and the Ancylus Lake were organic-poor clays (32). By circa 9,800 BP, a permanent gateway from the Baltic Sea to the North Sea was established, and the entire basin became a brackish-marine sea with high productivity (33), forming the Littorina Sea phase. Sediments deposited in the Littorina Sea and in the modern Baltic Sea were OM rich, highly reducing, and often methanogenic (27, 34). Overall, the contrasts in depositional conditions within the past ∼16,000 years (lacustrine versus marine, organic-rich versus organic-poor, etc.) create natural gradients that may influence the types and pathways of organic remineralization possible in the present-day microbial communities.

Previous studies in Baltic Sea sediments have shown that microbial communities vary between sediments deposited during different phases of the Baltic Sea (35). A recent metagenomic analysis of sediments recovered during the Integrated Ocean Drilling Project (IODP) Expedition 347 demonstrated significant differences in the microbial community structure and potential based on deposition, including halogenated compound degradation and C_1_ metabolisms, such as methane usage and the Wood-Ljungdahl pathway (36). A metatranscriptomics analysis concluded that microbes were active in Holocene-aged sediments up to 42 meters below seafloor (mbsf) (37). The focus of these studies were broad, and neither of these studies examined microbial organic matter mineralization potential in detail.

Here, we investigate sediments from several depths at four sites that differ in organic matter content and depositional histories, as follows: three sites within the Baltic Sea Basin and one site in the Kattegat body, the basin’s marine connection to the North Sea. We present new metagenomic sequencing data from Baltic Sea sediments and reanalyze previously published metagenomes and metatranscriptomes to determine which OM mineralization pathways are present in the sediments, assess which of these pathways are likely active, and connect these heterotrophic metabolisms to sediment facies and geochemistry.

## RESULTS

### Site description.

Sediment samples were collected from three locations in the Baltic Sea (sites M59, M63, and M65) and one location in the Kattegat region (site M60) (Fig. 1A). The water depths were between 31 m and 437 m, with cored sediment depths from 0.25 m to 67 mbsf (Table 1). Samples taken in the Baltic were deposited either under nonglacial conditions in the Holocene (samples M59E-0.25m, M59E-15m, M63E-12m, M65C-0.25m, M65C-3m, and M65C-10m) or during the Upper Pleistocene or Lower Holocene when the basin was under significant glacial meltwater influence, i.e., during the Baltic Ice Lake phase (samples M59E-67m, M63E-47m, and M65C-30m) (27). The nonglacial samples were deposited during marine phases (the modern Baltic Sea, the Littorina Sea, and the Yoldia Sea) or during the Ancylus Lake phase of the basin (see Fig. S1 in the supplemental material). The samples from M60B in the Kattegat region were marine in origin and were deposited in the Upper Pleistocene after deglaciation and marine transgression of the Kattegat (36).

**TABLE 1:** Sample locations and characteristics^*a*^

Site	Location	Seafloor depth (m)	Below seafloor depth (m)	Depositional conditions	Cl^−^-based salinity	Total carbon (wt%)	Total organic carbon (wt%)	Concn					
								Methane (mM)	Sulfate (mM)	Formate (μM)	Acetate (μM)	Propionate (μM)	Butyrate (μM)
59E Little Belt	55°0.285′N, 10°6.499′E	37.1	0.25	Holocene marine	23.30	6.40	5.40		0.25	1.61	3.95	0.96	0.17
			15	Holocene marine	24.23	5.95	4.97	1.13	0.07	10.38	20.54	4	0.43
			67	Glacial lacustrine	7.48	2.17	0.91	1.76	0.01	0.74	24.04	6.61	1.04
60B Kattegat	56°37.204′N, 11°40.229′E	31.2	24	Marginal marine	31.72	2.08	0.48	0.00	6.59	2.73	11.85	4.19	0.42
			37	Marginal marine	30.67	3.21	0.55	0.00	14.45	2.73	11.85	4.19	0.42
63E Landsort Deep	58°37.330′N, 18°15.240′E	437.1	11	Holocene marine	12.03	1.76	1.53	2.33	0.01	0.00	37.38	3.03	0.2
			47	Glacial lacustrine	1.67	0.70	0.55	9.40	0.02	0.38	22.73	4.71	0.14
65C Børnholm Basin	55°28.084′N, 15°28.624′E	84.3	0.25	Holocene marine	15	5	4.99	0.19	2.3	2.92	1.80	1.05	0
			3	Holocene marine	15.52	4.16	3.67	10.10	0.03	2.55	34.90	3.66	0
			10	Holocene lacustrine/marine transition	12.84	0.88	0.97	9.30	0.07	2.32	19.50	2.01	0.12
			30	Glacial lacustrine	2.78	2.48	0.48	0.80	0.22	0.00	22.80	2.46	0.25

a Location data, depositional conditions, salinity, total carbon, total inorganic carbon, alkalinity, methane, and sulfate data were originally published as part of the IODP Expedition 347 post-cruise report and by Andrén et al. (38), except for data for sample M65C-0.25m, which was published by Beulig et al. (39). Formate, acetate, propionate, and butyrate concentrations were measured in this publication, as detailed in Materials and Methods.

Total carbon (TC), total organic carbon (TOC), salinity, and methane were previously reported (27), and data specific to samples discussed herein are summarized here. The TC content of sediment in our samples varied between 0.70% dry weight (d.wt) in sample M63E-47m and 6.40% d.wt in sample M59E-0.25m (Table 1) (27). TOC content ranged from 0.48% d.wt in samples M60B-24m and M65C-30m to 5.40% d.wt in M59E-0.25m (Table 1 and Fig. 1C) (38). Methane was present in all samples except M60B-24m and M60B-37m. The highest measured methane concentration was 10.10 mM, which was in sample M65C-3m (Table 1) (27). Because the cores experienced substantial degassing during sampling, the reported values are minimum *in situ* methane concentrations (27, 34). Methane in these sediments is biogenic in origin (34, 39). Our Baltic samples (i.e., sites M59, M63, and M65) originated from within or below the main sulfate reduction zone, which is within the top meter of sediments, and the measured sulfate concentrations were therefore generally low (0.01 to 0.22 mM) (39) (Table 1). Samples from site M60 were the exception, with contained sulfate concentrations of 6.59 mM at 24 m and 14.45 at 37 m (Table 1) and no detectable methane until 92.88 mbsf (38). Generally, greater TOC and methane concentrations were observed in the nonglacial samples with high pore water salinities than in samples deposited under glacial conditions or during the Late Pleistocene. Oxygen was not measured in these samples, but *in situ* chemistry and previous studies indicate that sediments taken in the Baltic and Kattegat regions at these depths beneath the seafloor were anoxic (40, 41).

Pore water formate, acetate, propionate, and butyrate levels were measured (Table 1). Acetate was the most concentrated volatile fatty acid (VFA) measured, with concentrations ranging from 1.38 to 37.38 μM. Propionate concentrations ranged from 0.96 to 6.61 μM. Formate concentrations ranged from below the detection limit (BDL) to 10.38 μM. Butyrate was the least concentrated VFA, with concentrations ranging from BDL to 1.04 μM.

### Sequencing and assembly.

Coassembly of metagenomes presented here and metagenomes from a study by Marshall et al. (36) produced 557,851 contigs ≥1,000 bp in length. The coassembly contained a total of 1.07 Gb, with a maximum contig length of 159,111 bp and an average length of 1,923 bp (Table S1). Between 15.43% and 35.95% of metagenomic and metatranscriptomic reads in each sample mapped to the assembly. From the coassembly, 1,477,923 open reading frames (ORFs) were predicted (Table S1). Compared to all protein-coding genes in the InterProScan version 66.0, Pfam version 31.0, and TIGRFAM version 15.0 databases (accessed October 2017), based on sequence similarity, 1,074,069 ORFs were predicted to encode proteins within functional families or putative/hypothetical families in at least one of these databases. The remaining ORFs did not correspond to known or hypothetical genes in these databases.

### Protein utilization.

**FIG 2:**
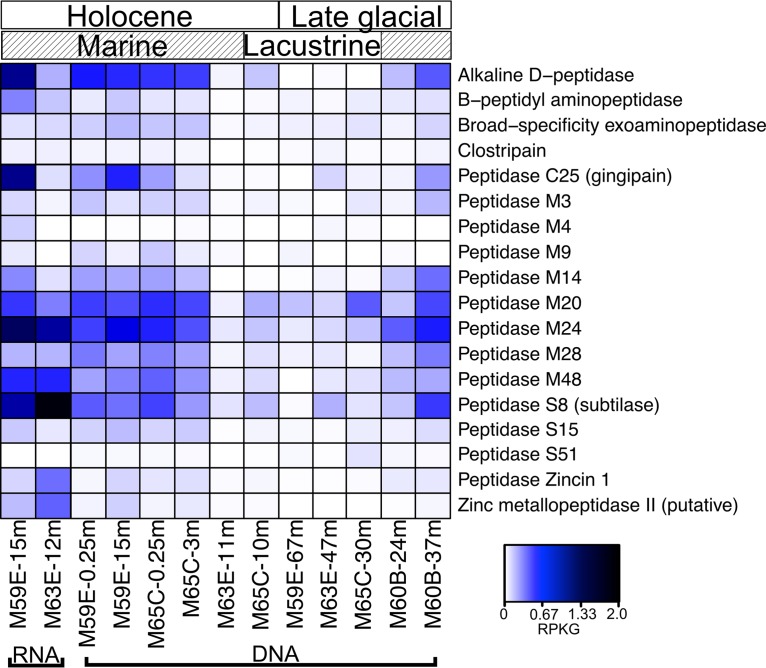
Abundances of ORFs which putatively encode extracellular peptidases (listed by MEROPS or Pfam nomenclature) in metagenomes and metatranscriptomes with heatmap color corresponding to RPKG. The type of peptidase putatively encoded in the metagenomes/metatranscriptomes is listed along the *y* axis. The *x* axis is arranged by sample type (RNA or DNA) and by the depositional times and environments. Along the top of the heatmap is the time period in which the samples were deposited (Holocene versus late glacial) and the state of the Baltic Sea or Kattegat (marine-influenced versus lacustrine). Marine-influenced samples were deposited during the Yoldia Sea or Baltic Sea phases of the basin or were deposited in the Kattegat body of water, which connects the North Sea and the Baltic Sea. Lacustrine-influenced samples were deposited during the Baltic Ice Lake or Ancylus Lake phases of the basin, when there was no significant influx of seawater to the basin.

Assembled ORFs were interrogated for sequences that were annotated as peptidase-encoding genes and contained an export signal peptide. ORFs predicted to code for peptidase families M24 (methionine aminopeptidase), S8 (subtilase), and M20 (glutamate carboxypeptidase) were the most abundant putative peptidase-encoding ORFs in total, with ORFs predicted to encode families C25 (gingipain) and M48 (Ste24 endopeptidase), which were also abundant across all samples (Fig. 2). Abundances of ORFs predicted to encode three of these peptidase families (glutamate carboxypeptidase, gingipain, and Ste24 endopeptidase) were significantly positively correlated with TOC content (linear regression, *P* < 0.05), and methionine aminopeptidase was significantly related to TOC content, salinity, and marine versus lacustrine depositional conditions (Fig. S2). The abundances of ORFs predicted to encode subtilase were not significantly associated with salinity or TOC content but were higher under marine than lacustrine depositional conditions (*t* test, *P* = 0.049), as were other less abundant putatively peptidase-encoding ORFs (Fig. S2). All metagenomic reads mapping to the predicted extracellular peptidase-encoding ORFs were significantly more abundant in samples with the greatest TC content (permutational multivariate analysis of variance [PERMANOVA], *P* = 0.007; Table S3). The relative abundances of these ORFs were also significantly positively correlated with salinity (*P* = 0.023), marine versus lacustrine depositional conditions salinity (*P* = 0.033), TOC content (*P* = 0.039), and formate concentrations (*P* = 0.021) (Table S3). The transcribed peptidase-encoding ORFs included those for alkaline d-peptidase, peptidase M24, gingipain, and subtilase (Fig. 2).

Phylum-level (or class level in the case of *Proteobacteria*) taxonomic assignment of the putative exported peptidase-encoding ORFs revealed sequences that were most similar to multiple bacterial and archaeal lineages (Fig. S3). These lineages included candidate phyla, such as “Candidatus Zixibacteria” and “Candidatus Omnitrophica,” as well as Bathyarchaeota, *Calditrichaeota*, *Planctomycetes*, *Alphaproteobacteria*, and Deltaproteobacteria. Mapping to these ORFs represented up to 16.8% of the total peptidase-encoding ORF abundance (Fig. S3). Most putatively exported peptidase-encoding ORFs were not confidently assigned taxonomy, including the ORFs with mapped transcripts.

### Carbohydrate utilization.

**FIG 3:**
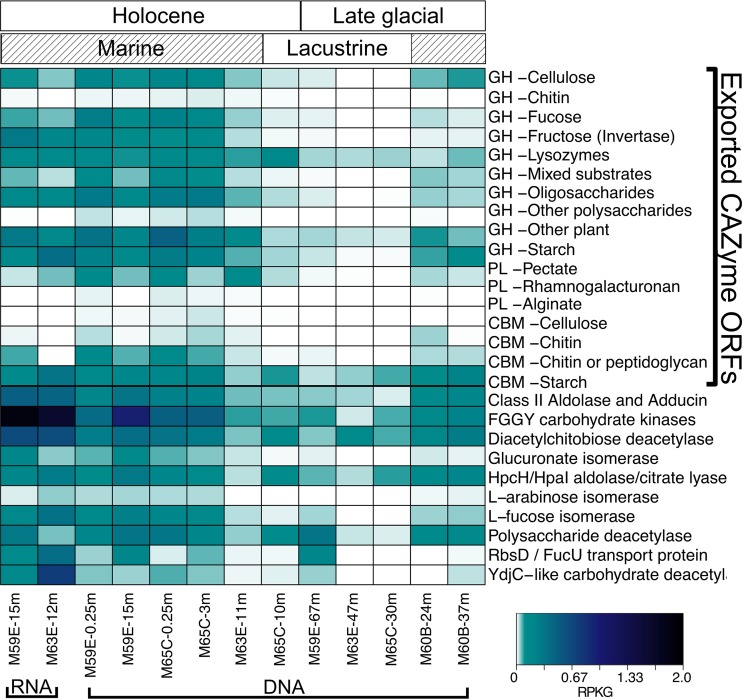
Abundances of ORFs which putatively encode carbohydrate degrading enzymes in metagenomes and metatranscriptomes with heatmap color corresponding to RPKG. The exported CAZyme ORFs bracket on the right side of the heatmap denotes putatively encoded CAZymes (glycoside hydrolases [GHs], polysaccharide lyases [PLs], and carbohydrate binding modules [CBMs]) with export signals and the target substrate(s). The organization of the samples follows Fig. 2.

ORFs annotated as coding for proteins that potentially mediate complex carbohydrate degradation (such as ORFs annotated as carbohydrate-active enzymes [CAZymes]) and that contained cellular export signals were examined. Glycoside hydrolases (GHs) are critical proteins in hydrolyzing complex carbohydrates (25). ORFs annotated as GH families with fucosidase, amylase, lysozyme, chitinase, cellulase, and xylanase activities were found (Fig. 3). Collectively, these GHs can degrade carbohydrates from various sources, including plants and algae (42). The most abundant ORFs potentially encoding exported GH families included families 5 (cellulase), 10 (xylanase), 23 to 25 (lysozymes), and 29 (fucosidase) (Fig. 3). The abundances of all of these ORFs annotated as encoding exported GHs except the lysozymes showed significant positive relationships with TOC content (linear modeling, *P* < 0.05) (Fig. S4). Furthermore, the abundances of putative exported CAZyme-encoding ORFs associated with plant matter, chitin, and starch degradation were significantly associated with marine versus lacustrine depositional conditions (Fig. S5). The abundances of ORFs annotated as encoding lysozymes were significantly correlated with marine Holocene versus glacially influenced depositional conditions (*t* test, *P* = 0.00015) (Fig. S4).

ORFs annotated as encoding exported carbohydrate binding modules (CBMs), which are associated with GHs and bind target substrates (43), were also found in this study. The most abundant ORFs annotated as encoding CBM found were mostly within families binding cellulose (family 10), chitin (family 5/12), and cell wall material, such as peptidoglycan (family 50) (44–46). Linear modeling showed that chitin-targeting CBM-encoding ORFs were significantly correlated with TOC content (*P* = 0.0002; Fig. S4). Other putatively exported CAZyme-encoding ORFs found included pectate lyases and alginate lyases, though alginate lyases were in low relative abundance (Fig. 3).

Permutation multivariate analysis of variance tests showed that total exported CAZyme-encoding ORF abundances corresponded most strongly with TOC (*P* = 0.009) (Table S3). These putative CAZyme-encoding ORF abundances showed weaker but significant correlations to salinity (*P* = 0.02), marine versus lacustrine depositional environment (*P* = 0.023), and approximate age of the sediments (*P* = 0.01) (Table S3).

The two metatranscriptomes showed that ORFs predicted to encode both GHs and CBMs were transcribed *in situ* (Fig. 3). Transcripts mapping to ORFs predicted to encode cellulases, fucosidases, invertases, lysozymes, plant matter (other than cellulose)-targeting GHs, and oligosaccharide-targeting GHs were relatively abundant, which was similar to the most abundant GH-encoding ORFs in the metagenomes. Of the transcribed CBM ORFs, chitin and peptidoglycan-targeting CBM ORFs were the relatively most abundant, with some transcripts mapping also to starch and plant matter-binding CBM-encoding ORFs (Fig. 3).

Putatively exported CAZyme ORFs were taxonomically assigned to many of the same lineages as the peptidases, such as the *Calditrichaeota*, “Candidatus Zixibacteria,” *Alphaproteobacteria*, *Planctomycetes*, and *Chloroflexi* (Fig. S3). Additionally, some CAZymes were assigned within the “Candidatus Lokiarchaeota,” and in one sample, M65C-0.25m, reads mapped to ORFs assigned within the bacterial candidate phylum “Candidatus Omnitrophica.” As with the peptidase ORFs, many ORFs were not able to be confidently assigned a taxonomy. The abundance of ORFs assigned within a phylum represented between 0 and 16.1% of the total putative exported CAZyme ORF abundance (Fig. S3). Transcripts mapped to ORFs assigned within *Chloroflexi*, “Candidatus Lokiarchaeota,” “Candidatus Zixibacteria,” Deltaproteobacteria, and *Planctomycetes* in M59E-15m and within *Chloroflexi* and *Planctomycetes* in M63E-12m (Fig. S3).

Microorganisms can hydrolyze complex carbohydrates into smaller molecules, which are then further metabolized intracellularly (16, 47). ORFs annotated as genes related to these processes were found in all samples (Fig. 3). In M59E-0.25m, M59E-15m, M59E-67m, M63E-11m, M65C-0.25m, and M65C-3m, most relatively abundant ORFs were assigned to the FGGY family, which is a broad family of carbohydrate kinases, such as gluconokinase, xylulokinase, fuculokinase, ribulokinase, and rhamnulokinase (48). ORFs annotated as transporter genes for fucose, which is a sugar subunit of the brown seaweed-produced polysaccharide fucoidan (49), were found in nonglacial metagenomes M59E-0.25m, M59E-15m, M63E-11m, M65C-0.25m, M65C-3m, and M65C-10m and were expressed in the metatranscriptomes but were absent in the glacial samples (Fig. 3). ORFs annotated as containing fucose isomerase genes showed a pattern similar to that of the fucose transporter genes (Fig. 3). ORFs annotated as encoding deacetylases were also found in all metagenomes and expressed in the metatranscriptomes. These included ORFs annotated as encoding diacetylchitobiose deacetylase, which contributes to chitin degradation by removing acetyl groups from diacetylchitobiose (50).

### Fermentation.

**FIG 4:**
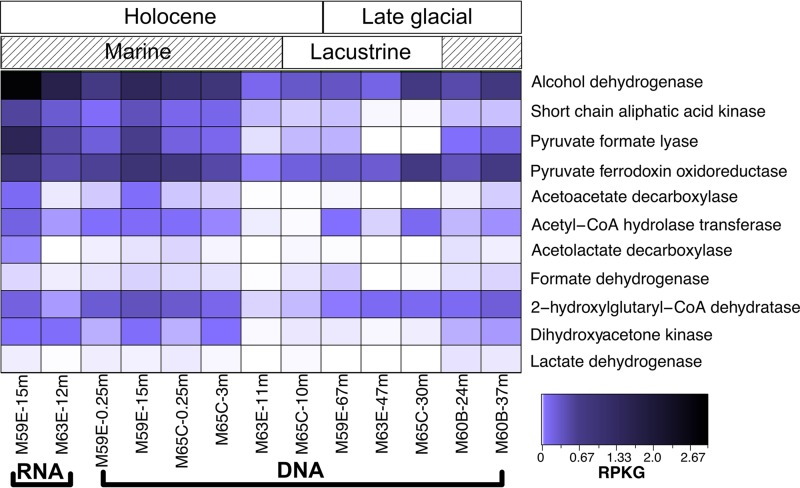
Abundances of ORFs which putatively encode fermentation-mediating enzymes in the metagenomes and metatranscriptomes with heatmap color corresponding to RPKG. The organization of the samples follows Fig. 2.

The metagenomes and metatranscriptomes were analyzed for ORFs encoding proteins putatively involved in fermentation. ORFs encoding proteins involved in pyruvate conversion to acetate were abundant, including both pyruvate ferredoxin oxidoreductase and pyruvate formate lyase, and these ORFs were mapped to by the two metatranscriptomic samples (Fig. 4). ORFs putatively encoding pyruvate ferredoxin oxidoreductase were more relatively abundant than ORFs putatively encoding pyruvate formate lyase in all metagenomes (*t* test, *P* = 0.0016) (Fig. 4). Putative pyruvate ferredoxin oxidoreductase-encoding ORF abundance was significantly correlated with TC content (linear modeling, *P* = 0.02) but not TOC content, and putative pyruvate formate lyase-encoding ORFs were significantly positively correlated with TOC content (*P* = 0.0035) and formate concentration (*P* = 0.00003) (Fig. S6). In the metatranscriptomes, these two genes were similarly abundant, with ORFs encoding pyruvate formate lyase slightly more abundant in each sample (Fig. 4). ORFs annotated as encoding acetyl-coenzyme A (acetyl-CoA) hydrolase/transferase, which facilitates the production of acetate from acetyl-CoA or the production of acetyl-CoA from acetate and acyl-CoA, and acetate kinases were found in all samples. Acetate kinase-encoding ORF abundances were significantly correlated with TOC content (linear modeling, *P* = 0.00025), formate concentrations (*P* = 0.00012) (Fig. S6e and f), and salinity (*P* = 0.00016).

ORFs annotated as coding for alcohol dehydrogenases, which are reversible enzymes that can produce alcohols, such as ethanol, during fermentation (51, 52), were also present and abundant in all samples (Fig. 4). In nonglacial samples, the relative abundance of ORFs annotated as encoding alcohol dehydrogenases was greater than the relative abundance of ORFs encoding pyruvate targeting proteins (Fig. 4). Putative alcohol dehydrogenase ORF abundances were significantly correlated with TC content (linear modeling, *P* = 0.0022; Fig. S6).

Taxonomic assignments of fermentation ORFs were within diverse phyla, including the *Chloroflexi*, *Planctomycetes*, *Alphaproteobacteria*, Deltaproteobacteria, “Candidatus Omnitrophica,” “Candidatus Lokiarchaeota,” *Calditrichaeota*, and Bathyarchaeota (Fig. S3). These ORFs represented 0 to 35.2% of the total reads mapped for fermentation genes. Transcripts mapped to fermentation ORFs assigned within *Chloroflexi*, “Candidatus Lokiarchaeota,” and *Calditrichaeota* from both metatranscriptomes. Additionally, M59E-15m transcripts mapped to ORFs assigned within Bathyarchaeota, and M63E-12m transcripts mapped to ORFs assigned within *Planctomycetes* (Fig. S3).

Other present and expressed putatively fermentation-related ORFs included 2-hydroxylglutaryl-CoA dehydratase-encoding ORFs (Fig. 4). 2-Hydroxylgluatryl-CoA dehydratase catalyzes a key step in glutamate fermentation (53, 54). ORFs potentially encoding proteins that oxidize glycerol, dihydroxyacetone kinase, and glycerol dehydratase (55) were found in low abundances in the metagenomes, and at least one of these putative ORF types was expressed in each metatranscriptome. ORFs putatively encoding acetoacetate decarboxylase, which produces acetone and CO_2_ from acetoacetate during fermentation (56), were present in all Holocene metagenomes (Fig. 4), and their abundances were strongly correlated with TOC content (*P* = 0.0042). The overall abundance of putative fermentation ORFs in samples was significantly related to TOC content (PERMANOVA, *P* < 0.05) but not to salinity, marine versus lacustrine sediment deposition, any fatty acid examined here, or sediment age (Table S3), and it generally did not show strong grouping by sample type with clustering analyses (Fig. S7c).

## DISCUSSION

### Organic matter degradation.

Protein-derived compounds account for >20% of OM in some surface sediments (57, 58). In organic-rich sediments, the microbial genetic potential to degrade macromolecules has previously been demonstrated through genomic analyses (26, 59, 60). In our study, putative peptidase-encoding ORFs with export signal sequences were abundant in marine samples (Fig. 2). The relative abundance of ORFs putatively encoding peptidases was positively correlated with sediment characteristics, including TOC content, salinity, and marine versus lacustrine depositional conditions (Table S3). Recently, Schmidt and Steen incubated Baltic Sea sediment with labeled peptidase substrates and showed that extracellular peptidases from site M59 were active down to 55 mbsf in the organic-rich Holocene sediment (61). Without further information about the origin of the ORFs, such as metagenomic binning or single-cell genome amplification, taxonomic assignments of ORFs are solely based on similarity to previously characterized gene sequences. However, the suggested taxonomies of some of the putative extracellular peptidase-encoding ORFs are similar to those found previously in marine sediments. For example, some peptidase-encoding ORFs here were assigned within the *Bathyarchaeota* or *Calditrichaeota* phyla. Archaeal genomes isolated from *Bathyarchaeota* from Aarhus Bay at the entrance to the Baltic Sea contained peptidases, including gingipain and clostripain, which also appeared to be active in enzyme assays of whole sediment (62). Marshall et al. (63) showed that members of *Calditrichaeota*, a bacterial phylum found in the Baltic Sea and in marine sediments globally, also likely degrade extracellular proteins for energy (63). These studies support our findings that both bacterial and archaeal lineages commonly found in marine sediments could mediate peptide degradation in Baltic Sea sediments here.

Our findings are also similar to what was observed in metatranscriptomes from Peru Margin sediment, where transcripts predicted to be extracellular peptidases were abundant and taxonomically assigned to archaeal lineages, including *Bathyarchaeota* (59). While it is not possible to determine the *in situ* metabolic capabilities or activities of the microbial communities based on gene predictions alone, the results here and previous findings support the idea that protein degradation for energy acquisition is an important heterotrophic strategy in the marine deep biosphere and, based on metatranscriptomics, was likely active in some of these sediments.

Globally, near-shore environments receive 0.4 pg year^−1^ of riverine OM, including structural parts of vascular plants composed mainly from lignin, cellulose, and xylan (64). Our analyses here indicate that ORFs predicted to encode CAZymes associated with plant matter degradation were abundant in Baltic Sea sediments, including multiple glycoside hydrolases and carbohydrate binding modules (Fig. 3). The abundances of these genes were positively correlated with TOC content (Fig. S4 and Table S3), though it is unknown what proportion of TOC is bioavailable to microbial activity or how it varies between sediment samples. Many CAZyme genes were transcribed in Baltic sediments at 12 and 15 mbsf, suggesting active plant matter degradation (Fig. 3). CAZyme-encoding genes have been found in estuarine sediments (60, 65), in deep-sea sediments (66), and in the top 10 cm of sediment from the Landsort Deep (Baltic Sea) (67). Recently, genes encoding proteins that mediate plant-derived OM degradation were found transcribed in Peru Margin sediments (59). Our results demonstrate a genetic potential for plant matter degradation deep into Baltic sediments and that these genes are actively transcribed to depths of at least 15 mbsf.

**FIG 5:**
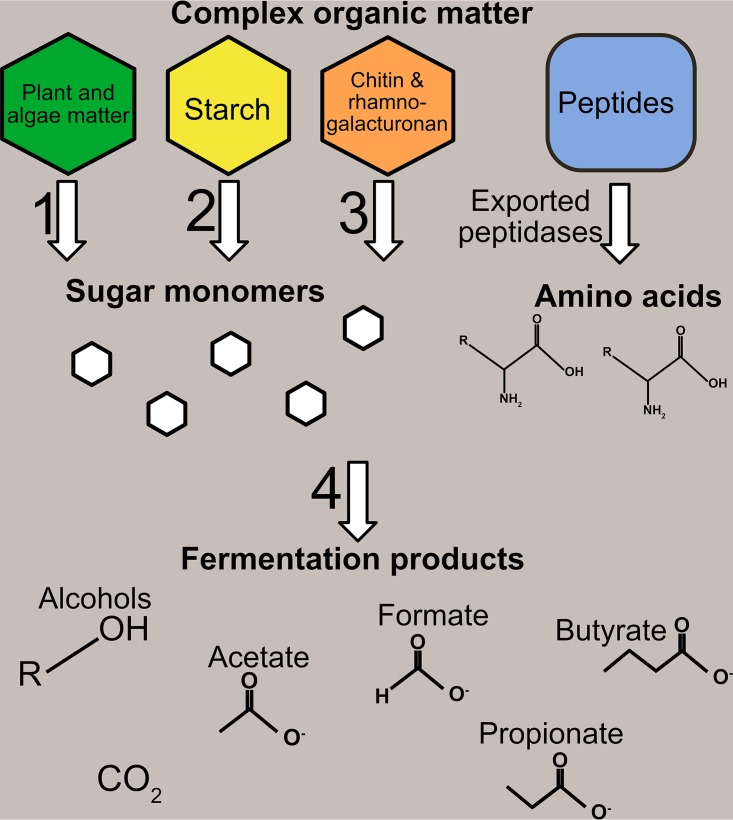
Schematic overview of OM degradation pathways investigated in this study. Assignments of potential ORFs involved in the arrow numbers: 1, fucose, cellulose, and other plant matter GHs, pectate and alginate polysaccharide lyases, cellulose and starch CBMs, glucodextranase, FGGY carbohydrate kinase, l-arabinose isomerase, l-fucose isomerases, pectinesterase, glucuronate isomerase, aldolases, fucose transporters, and polysaccharide deacetylase; 2, starch GHs, starch CBMs, and alpha-amylase/4-alpha-glucanotransferase; 3, chitin GHs, rhamnogalacturonan PLs, chitin CBMs, YdjC-like carbohydrate deacetylase, and diacetylchitobiose deacetylase; 4, ORF assignments from Fig. 4.

Macroalgae are primary producers which can rapidly produce biomass, which is then deposited in coastal sediments (up to 3 kg C m^−2^ year^−1^) (68). In Holocene sediments examined here, putative alginate lyase-encoding ORFs were found in low but detectable numbers at sites M59E, M63E, and M65C and were expressed in M63E-12mbsf and 59E-15mbsf. However, they were not found in sites associated with glacial conditions, which were deposited before the major postglacial establishment of brown algae in the Baltic (69). Brown algae are typically marine seaweeds which produce fucoidan (70). ORFs putatively encoding fucosidases were abundant in sediments deposited during marine-brackish periods, and these ORFs were expressed in the metatranscriptomes. Similarly, we found that ORFs putatively encoding chitinase, which breaks down chitin (71–73), and chitin-binding CBMs were more abundant in samples deposited during periods of marine inputs. Chitin is a major component of the exoskeleton of arthropods and is abundant in marine systems (74). It short, it appears that diverse microbial potential for OM degradation is present in Baltic Sea sediments (Fig. 5). However, due to uncertainties in omics-based studies, experimental evidence is needed to verify that these communities are able to carry out these suggested activities.

### Fermentation potential.

ORFs putatively related to fermentative metabolism were ubiquitous in all samples here, were likely taxonomically diverse (Fig. S3), and included those mediating anaerobic fermentation of various substrates (Fig. 4). Particularly abundant were ORFs which putatively confer the ability to break down pyruvate into acetate, i.e., pyruvate ferredoxin oxidoreductase and pyruvate formate lyase. In addition to being among the most abundant genes in the metagenomes, they were also present in the two metatranscriptomes (Fig. 4). These observations agreed with our findings that acetate concentrations in the sediments were higher than those of the other VFAs measured here (Table 1), though neither of these ORF abundances significantly correlated with acetate concentrations. This could be due to the control of VFA turnover by consumers as opposed to producers of VFAs, i.e., fermenters (75).

Alcohol dehydrogenase-encoding ORFs were similarly abundant as ORFs putatively encoding acetate-producing proteins in the metagenomes and were more abundant in the two metatranscriptomes (Fig. 4). In bacteria, archaea, and the yeast *Saccaromyces cerevisiae*, alcohol dehydrogenase is usually a reversible enzyme, which can produce or break down various alcohols, such as ethanol (51, 52, 76–78). Though we were unable to determine if the dominant function here was alcohol production or consumption, these results indicated that alcohol turnover was an important aspect of carbon cycling in the sedimentary subsurface. Sediments from other marine environments, including methane-rich cold seeps, also have an abundance of alcohol-producing genes (79). Direct measurements within the top meter of Gulf of Mexico sediments revealed methanol and ethanol concentrations up to 69 μM and 43 μM, respectively (80). Furthermore, laboratory studies of fermentation occurring in sediments collected from tidal flats found that ethanol was the second most concentrated fermentation product (acetate was the most concentrated) for at least the first week of the experiment (81). In light of these findings and the recent development of a method to detect micromolar amounts of methanol and ethanol from marine sediments (80), future studies of sediment alcohol dynamics are warranted.

### Impacts of depositional condition on microbial community function.

The deglaciation of the Baltic Sea Basin during the late Pleistocene and fluctuating water column conditions throughout the Holocene are reflected in the lithology and *in situ* geochemistry of Baltic Sea sediment (38). We observed major differences between marine and lacustrine, and organic-rich and organic-poor sediments in the types of possible carbon catabolism, including carbohydrate degradation and extracellular protein degradation. The abundances of these OM degradation ORFs generally were higher in the high organic nonglacial sediments than in the glacial samples (Fig. 2 and 3). However, gene abundances at site M60B, most notably within the putative CAZyme- and peptidase-encoding ORFs, often did not follow the correlation between TOC content and ORF abundances (Fig. 2, 3, and S7a and b). Site M60B is located outside the Baltic Sea Basin in the Kattegat region, and the samples examined here from 24 and 37 mbsf were deposited during the Kattegat deglaciation ca. 15,900 to 16,500 years BP (82). During this period, the Kattegat water column was marine-brackish due to inputs from the North Sea and glacial meltwaters (82), whereas the Baltic Sea Basin was cut off from marine inputs.

One explanation for the abundances of putative CAZyme- and peptidase-encoding ORFs in site 60B could be the depositional history. Most of the marine-influenced samples were from the Holocene (M59E-0.25m, M59E-15m, M65C-0.25m, M65C-3m, and M63E-11m), but the marine-influenced M60B samples were deposited during the Late Pleistocene as the Scandinavian Ice Sheet was retreating (27). During the Late Pleistocene, site M60 received inputs from both glacial meltwater runoff and from the North Sea, and microfossil analyses indicate significant deposition of terrestrial material from glacial meltwater, including Cretaceous-age microfossils (38). Pollen analyses have shown that the easily degradable pollen was underrepresented in the M60B samples relative to pollen that is more resistant to degradation. This was interpreted as extensive degradation of the pollen *in situ*. This suggests that OM mineralization was active in these sediments postdeposition, and geochemistry indicates that some microbial activity is still occurring *in situ* (38). In contrast, the other glacially influenced samples were deposited during times with low-water-column productivity and show little evidence of current *in situ* OM remineralization, as indicated by *in situ* geochemistry (Table 1). In short, it appears that the depositional conditions influenced modern geochemistry, such as the amount of carbon in sediments, which influences present-day abundances of microbial community genetic potential to break down macromolecules for metabolic use.

The finding that depositional conditions influence microbial community organic matter degradation potential highlights previous observations not only in Baltic Sea sediments (35, 36), but in Arabian Sea sediments as well (83). Sediments in the Arabian Sea, like in the Baltic Sea, exhibit an order of magnitude variation in TOC content corresponding to climate fluctuations over a glacial-interglacial cycle (83). Metagenomic analysis revealed that microbial protein degradation genetic potential was significantly correlated with TOC content in the interglacial sediments in Arabian Sea sediments (83). Similarly, lacustrine sediments from Laguna Potrok Aike, Argentina, were deposited under varied salinity and water column productivity during the last glacial-interglacial cycle (84). Based on 16S rRNA gene profiling, sediment microbial communities were found to vary significantly with these parameters, and it was concluded that climate-related depositional conditions played a role in shaping the subsurface microbial community (84). The culmination of these studies and others (85) with the current study here indicates that depositional conditions and the associated geochemical or lithological conditions impact sediment microbial communities and their metabolic potential postdeposition in a variety of aquatic environments.

In summary, we examined microbial community genetic potential and, in two samples, transcriptional activity to determine the types and relative abundances of microbial heterotrophy in Baltic Sea Basin sediments. We determined that in the organic-rich sediments, there was genetic potential for multiple metabolic strategies, including protein degradation, complex carbohydrate usage, and fermentation (Fig. 5). Furthermore, based on metatranscriptomic analyses, these pathways were active in the two samples. Fermentation potential, including the potential for alcohol production, was ubiquitous in all sediments examined. Finally, the abundance patterns of carbohydrate-active-enzyme- and peptidase-encoding genes indicated that both *in situ* and sediment depositional conditions are important in determining the types of organic mineralization potential present.

## MATERIALS AND METHODS

### Sample collection.

Samples M59E-15m, M59E-67m, M60B-24m, M60B-37m, M63E-11m, M63E-47m, M65C-3m, M65C-10m, and M65C-30m were collected by the Integrated Ocean Drilling Program (IODP) aboard the MSP *Greatship Manisha* in September to November 2013. The sediment cores were taken by advanced piston coring. On board, cores were cut into 1.5-m sections, sampled for perfluorinated chemical (PFC) contamination (to assess the amount of drilling disturbance), scanned with a fast-track multiple-scanning core logger, and sectioned into whole round cores in a 12°C microbiology container onboard the ship. Sediment cores for nucleic acid analyses were immediately frozen at −80°C on ship and shipped to land-based laboratories on dry ice (cf. reference 38).

To sample the top few meters of sediment, which were not recovered in the drilling process, two cruises lead by the Center for Geomicrobiology at Aarhus University on the R/V *Aurora* were undertaken. In September 2014, site M59 was revisited. Sample M59E-0.25cm was collected through gravity coring, followed by subsampling through windows cut into the sediment core liner and the insertion of sterile 20-ml syringes with the ends cut off. In June, 2016, site M65C was revisited, and sample M65C-0.25m was collected through Rumohr coring (as in reference 39). Subsampling was similar to that with sample M59E-0.25m, in which sterile cut-off 20-ml syringes were used to subsample the 25-cm-depth horizon. Care was taken to avoid potential seawater contamination by visually inspecting the cores for seawater intrusion and extracting DNA from sediment collected from the interior of the core. All samples were immediately frozen at −80°C on ship and were shipped to the United States on dry ice.

### Volatile fatty acid analysis.

Pore water samples for volatile fatty acid (VFA) analysis were retrieved with Rhizon soil moisture samplers (Rhizosphere Research Products, Wageningen, The Netherlands) (86) or were obtained by a hydraulic press (87) according to IODP standard protocols if the sediment was to compacted. Rhizon samplers were precleaned with 50 ml Milli-Q water (ultrapure, type 1) and stored in vacuum-sealed gas-tight bags (24). The samples were stored at −80°C in 4-ml borosilicate glass vials (Zinsser Analytic, Germany) that were previously baked for 5 h at 450°C. Prior to the analysis, the samples were defrosted and filtered through disposable Acrodisc 13-mm ion chromatography (IC) syringe filters (pore size, 0.2 μm) that were rinsed with 10 ml Milli-Q (ultrapure, type I) water directly before use. The first 0.5 ml of pore water after filtration was discarded, and a second 0.5 ml was used for analysis. VFA concentrations, including formate, acetate, butyrate, and propionate, were measured by two-dimensional IC mass spectrometry (2D IC-MS), as described in detail by Glombitza et al. (24). Briefly, in this method, the first IC dimension was used to separate inorganic ions, such as chloride, from VFAs. VFAs were trapped on a concentrator column and subsequently separated in the second IC dimension. Quantification was achieved by the mass spectrometer in the single ion monitoring (SIM) mode. The detection limits were 0.37 μM for formate, 0.19 μM for acetate, 0.12 μM for propionate, and 0.09 μM for butyrate. Quantification was achieved by a 3-point calibration with external standards of a mixture of VFAs (formate, acetate, and propionate) at different concentrations (i.e., 200, 500, and 800 μg liter^−1^) in an International Association for Physical Sciences of the Ocean (IAPSO) seawater standard (Ocean Scientific International Ltd. [OSIL], UK).

Other geochemical data for IODP cores (all samples here except M59E-0.25m and M65C-0.25m) were collected and analyzed as described in reference 38. Briefly, Rhizon soil moisture samplers and core squeezers were used to retrieve sediment pore water. Sulfate and Cl^−^ were measured via ion chromatography using a Metrohm 882 compact ion chromatograph (Herisau, Switzerland) at the University of Bremen. Methane samples were collected from fresh core material, extruded into 8 ml of 1 M NaOH-filled glass vials, shaken and equilibrated, and measured on an A7890 gas chromatograph (Agilent Technologies, Santa Clara, CA, USA) (34, 38). TC and TOC were sampled from 10 cm^3^ of freeze-dried and ground sediment. TC measurements were derived from approximately 65 mg of sample that was combusted, and evolved CO_2_ was measured on a CS-300 carbon-sulfur analyzer (Leco Corporation, St. Joseph, MI, USA). TOC was measured from 65 mg of 12.5% HCl decalcified sediment, which was then heated, and evolved CO_2_ was measured as described above (38).

For samples M59E-0.25cm and M65C-0.25cm, geochemical analyses were performed as described previously (39). Rhizon samplers extracted sediment pore water, which was then acidified and measured at the University of Aarhus (88). Methane samples were collected immediately after core retrieval, transferred into vials with 4 ml of saturated NaCl, capped, stored at −20°C, and measured on an SRI 310C gas chromatograph equipped with an SRI 310C flame ionization detector (SRI Instruments, Torrance, CA, USA) (39).

### Extraction.

DNA from samples M59E-0.25m, M59E-15m, M65C-0.25m, M65C-3m, and M65C-10m was extracted from sediment using the DNeasy PowerMax soil kit (Mo Bio Laboratories, Carlsbad, CA). Frozen sediment was chipped from whole round cores (M59E-15m, M65C-3m, and M65C-10m) or cut-off syringes (M59E-0.25m and M65C-0.25m) in a dedicated clean room at Texas A&M University Corpus Christi. All instruments used were treated with ethanol and flame-sterilized, and the edges of the sediment core were avoided during collection. Researchers wore face masks and hair nets to avoid sample contamination. Between 5 and 10 g of sediment was extracted for each sample. The manufacturer’s protocol was followed, including the final concentration step and resuspension of DNA in 100 μl of molecular-biology-grade water. Sample-free negative controls (kit blanks) were processed and sequenced alongside the samples. These negative controls were below the detection limit (0.5 ng DNA μl^−1^) when measured using the Qubit DS high-sensitivity kit and did not amplify when subject to PCR of the 16S rRNA gene and visualized on a 1% agarose gel.

### Sequencing.

Metagenomes from samples M59E-0.25m, M59E-15m, and M65C-0.25m were sequenced at the Marine Biological Laboratories (Woods Hole, MA, USA). Metagenomic library preparation and sequencing followed the Census of Deep Life protocol as described by Vineis et al. (89). The modifications to the Vineis protocol were that the sequencing platform was the NextSeq (Illumina, San Diego, CA, USA), which produced 150-bp-long paired-end reads, and no microbiome enrichment step was conducted. The insert sizes were 170 bp.

Metagenomes from samples M65C-3mbsf and M65C-10mbsf were sequenced at the Research and Testing Labs (RTL; Lubbock, TX, USA). Libraries were prepared using the HyperPlus kit (Kapa Biosystems, Wilmington, MA, USA), according to the manufacturer’s instructions, with the following modifications: ligation was increased to 1 h at room temperature, postligation cleanup used 0.75× beads, and the postamplification cleanup bead concentration was increased to 0.7×. Libraries were sequenced on the HiSeq 2500 system (Illumina, San Diego, CA, USA), producing 150-bp-long paired-end reads. The insert sizes were 170 bp.

See Table S1 for sequencing statistics. Note that the metagenome M63E-12m and the metatranscriptome M63E-12m were both extracted from sediment core M63E-6H2 (IODP nomenclature), which spanned from 10 to 12 mbsf (36–38). To maintain consistency with these prior publications, the names of the metagenome and metatranscriptome from this core used in this publication are M63E-12m and M63E-11m, respectively. The differences in sequencing coverages between the metagenomes presented here and the previously published metagenomes could cause some bias in detection of low-abundance ORFs. Additionally, assembling these samples individually was not ideal due to the low sequencing coverage in the samples from the study by Marshall et al. (36), making coassembly of all metagenomes the optimal choice and consistent with previous studies (90–92).

### Bioinformatics. (i) Quality control and assembly.

Reads were trimmed using the program Trim Galore! version 0.4.3 (Babraham Bioinformatics, Cambridge, United Kingdom) in paired-end read mode, with a minimum quality score of 25, a maximum of 4 low-quality bases before the read was trimmed, and the read length must be a minimum of 80 bp long posttrimming. Samples were deduplicated using Super Deduper version 2.0, with default settings (starting location of 10 bp, 25 bp in the unique identification [ID]) (93). All metagenomes were coassembled using MEGAHIT version 1.0.3-29-g707d683, with a minimum contig size of 1,000 bp (94, 95). Default k-mer sizes of 21, 29, 39, 59, 79, 99, 119, and 141 were used for assembly. Contig names were simplified using anvi-script-reformat-fasta in anvi’o v2.4.0 (96).

### (ii) Read mapping and profile generation.

Metagenomic and metatranscriptomic reads were mapped to the assembled contigs using Bowtie2 version 2.2.5 using the “sensitive” end-to-end setting (97). The resulting .sam files were converted to bam files using SAMtools version 1.5, and these files were converted to anvi’o-compatible .bam files in anvi’o. An anvi’o database was created from the contigs, which included ORF determination using Prodigal (98). Each sample was profiled against the contig database using the anvi’o command anvi-profile. A full project database was constructed from these profiles and included information about gene coverage and detection (percentage over which the ORF was mapped by reads). Tables with gene coverage by sample and gene detection by sample were exported using the anvi’o command anvi-export-gene-coverage-and-detection. In this command, gene coverage is reported as coverage of each base pair of the gene from mapped reads, divided by the length of the gene, and these values were then normalized to reads per kilobase per billion (RPKG). Only genes with at least 50% detection, referring to at least 50% of the gene being represented by at least 1× coverage prenormalization, were considered (96).

### Taxonomic and function assignments of genes.

ORFs were exported as amino acid sequences from the contigs for taxonomic and functional assignment using the anvi’o command anvi-get-aa-sequences-for-gene-calls. Function was assigned to assembled genes using InterProScan version 5.26-65.0 (99) against the Pfam version 31.0 (100) and TIGRFAM version 15.0 databases (101) (accessed September 2017) using the precalculated lookup service. Functional assignments were exported as a tab-separated file and parsed in R. Genes annotated as peptidase or CAZyme coding were screened for export peptides using SignalP targeted for bacteria (Gram positive and negative) and eukaryotes (102), and were screened using PSORTb targeted for archaea (103). Protein families of interest were manually searched in the Pfam database in August to November 2017. Pathway reconstruction was based on pathways in the KEGG (104) and Metacyc databases (105) and published literature. Putative peptidase-encoding gene functions were described using the MEROPS peptidase nomenclature (106). Glycoside hydrolase functions were inferred from the Pfam GH assignment using the functions as described in the CAZYpedia and in published literature (25, 42). These functions were summarized by putative substrate in Fig. 3. For a complete list of genes examined and their Pfam numbers, see Table S2.

ORF sequences were compared to the NCBI nonredundant database (accessed December 2016) using the blastp mode of DIAMOND version 0.8.36 (107) with “sensitive” setting and allowing only one match per sequence allowed. The DIAMOND results were uploaded to MEGAN version 6.10.2, and taxonomy was assigned using the weighted lowest common ancestor (LCA) assignment algorithm with a minimum support percent identity of 0.3 (i.e., a taxon must have at least 0.3%, or 2,888 ORFs, assigned to be considered a “real” hit, as suggested for mixed communities in the user manual) (108, 109). Taxonomic assignments for ORFs were exported as a tab-separated file and parsed in R.

### Statistical analyses.

All statistics, including linear modeling, canonical correspondence analyses, Student’s *t* tests, and permutational analysis of variance (PERMANOVA), were performed using the vegan package version 2.4-4 (110) in R version 3.4.2, and the type of statistical test used is indicated in the text. For tests involving geochemical parameters, such as salinity or TOC content, values listed in Table 1 were used. Sediment age was estimated from previously published scientific literature (111, 112). Marine versus lacustrine conditions were defined by the stage of the Baltic Sea Basin during sediment deposition, i.e., Baltic Ice Lake and Ancylus Lake were lacustrine and the Yoldia Sea, Littorina Sea, and the modern Baltic Sea were marine (Table 1 and Fig. S1). Both Kattegat samples were marine. All *P* values reported herein were determined to be statistically significant at a value of less than 0.05. The ggplot2 package (113) was used to create graphics.

We recognize that this data set is a relatively small sample set (*n* = 11), and this may reduce the robustness of statistical analyses. The application of statistical analyses nevertheless has merit even in environments of low sample density and has previously been applied successfully in the marine deep biosphere (see, e.g., references 35 and 61). The variation in sequencing coverage across each sample could bias results by underestimating low-abundance genes in samples sequenced with lesser coverage, and our coassembly could be biased towards the samples that contained a greater abundance of sequences. However, our results consistently indicated that the abundance and type of OM degradation potential found in the metagenomes were related to several sediment characteristics, including TOC content and depositional facies.

### Data availability.

Metagenomic data for M59E-0.25m, M59E-15m, M65C-0.25m, M65C-3mbsf, and M65C-10mbsf can be found in the National Center for Biotechnology Information (NCBI) under BioProject number PRJNA433242. Metagenomes from all other samples here were sequenced as described by Marshall et al. (36) and were retrieved from the NCBI SRA project SRP068645. Metatranscriptomes were sequenced as described by Zinke et al. (37) and archived under the NCBI SRA project SRP108285.

## Supplementary Material

Supplemental file 1
