# Supplementary material for: Microbial Organic Matter Degradation Potential in Baltic Sea Sediments Is Influenced by Depositional Conditions and In Situ Geochemistry
Source: Appl Environ Microbiol. 2019 Feb 6;85(4):e02164-18. doi: 10.1128/AEM.02164-18 (PMC6365825; doi:10.1128/AEM.02164-18)
Supplement: Supplemental file 1 [file d2bfe4c05401469e11a6ff06ed5920df_AEM.02164-18-s0001.pdf]

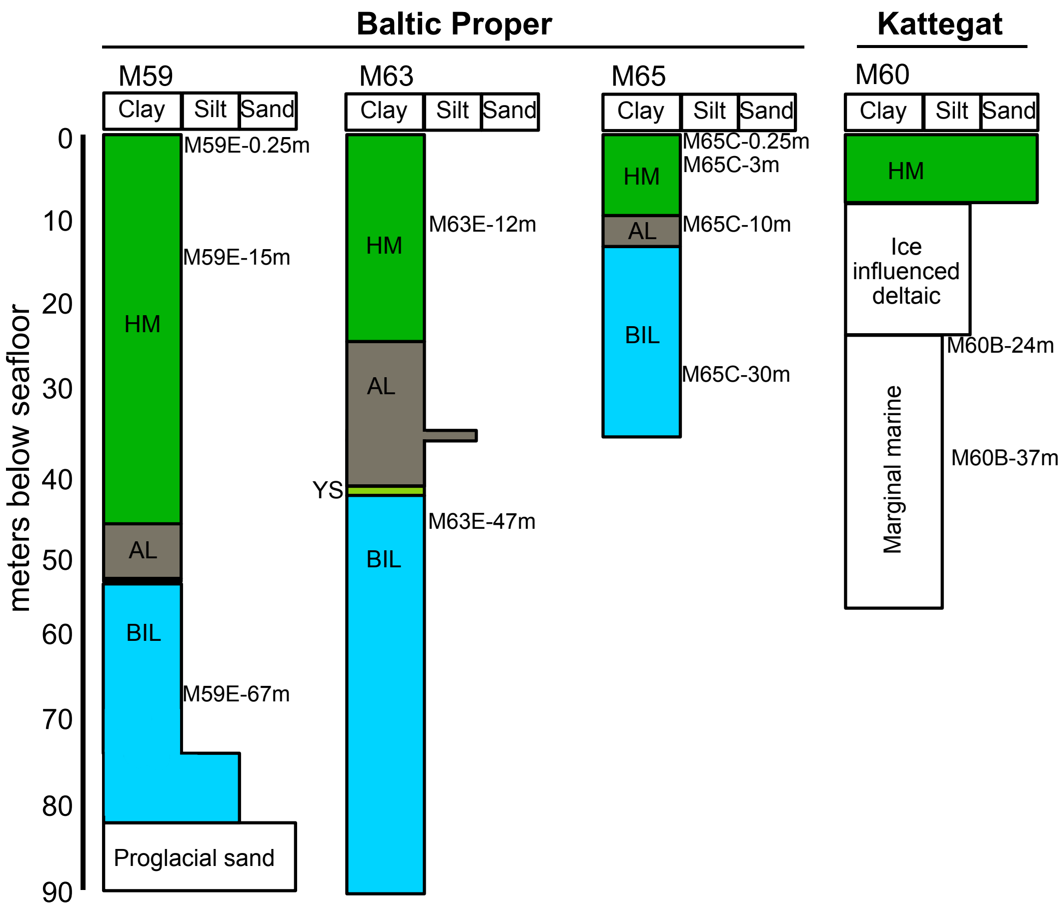

Supplemental Figure 1. Schematic of sediment core profiles and characteristics. Samples are labeled to the right of the sediment column. HM: Holocene Mud (which represents both the Littorina Sea and modern Baltic Sea); AL: Ancylus Lake; YS: Yoldia Sea; BIL: Baltic Ice Lake.

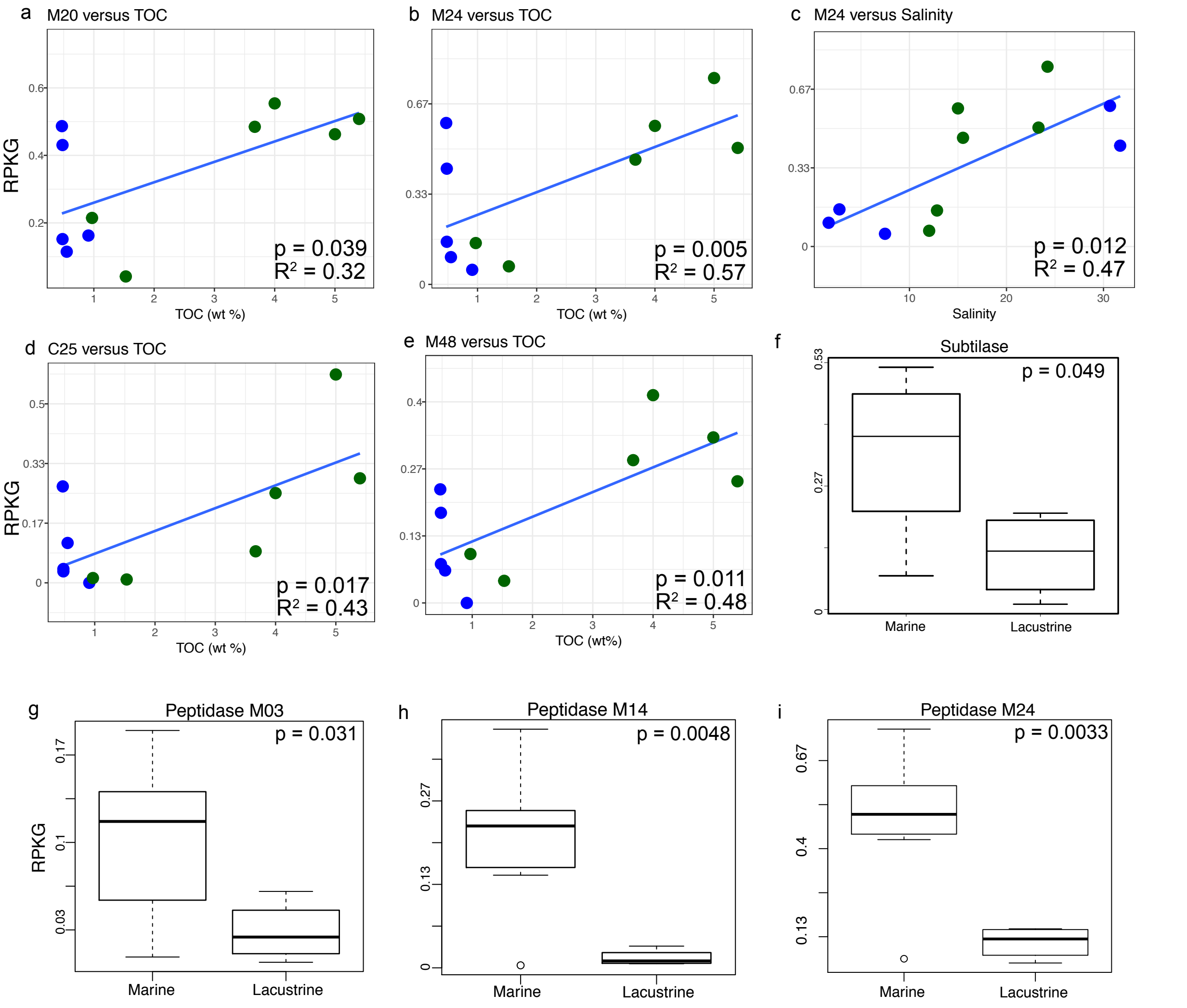

Supplemental Figure 2. Linear models (a-e) or boxplots (f-i) of abundant ORFs putatively encoding extracellular peptidases compared to select environmental parameters. Blue dots represent samples deposited during the late Pleistocene and are most glacially influenced, green samples were deposited during the Holocene (i.e. the Ancylus Lake, Littorina Sea, and Baltic Sea phases of the Baltic's history). P-values for boxplots are based on t-tests.

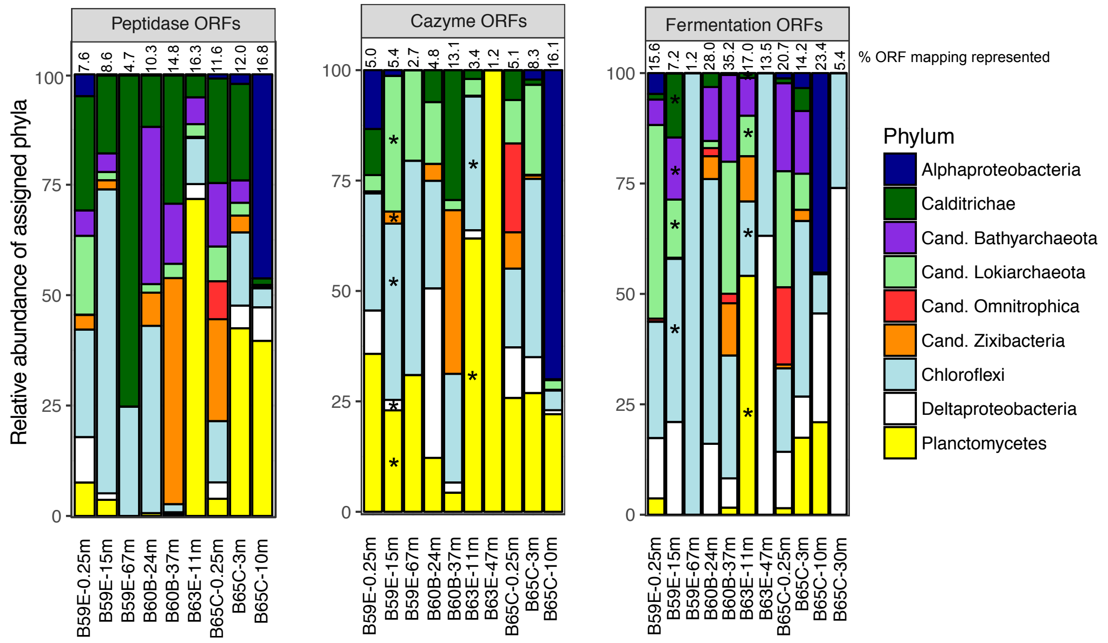

Supplemental Figure 3. Taxonomic assignments at the phylum level of ORFs putatively encoding CAZymes, peptidases, or fermentation proteins. Relative abundance on the y-axis represents the coverage of contigs assigned to each phylum relative to the coverage of ORFs which were assigned taxonomy at the phylum level by the least common ancestor algorithm in MEGAN. These relative abundances only include abundances of ORFs which were assigned within a phylum, and the percent of coverage for represented for that ORF category is shown along the top of the bars. Only samples with mapped ORFs assigned within a phylum were graphed here. Asterisks represent phyla with mapped metatranscriptomic reads.

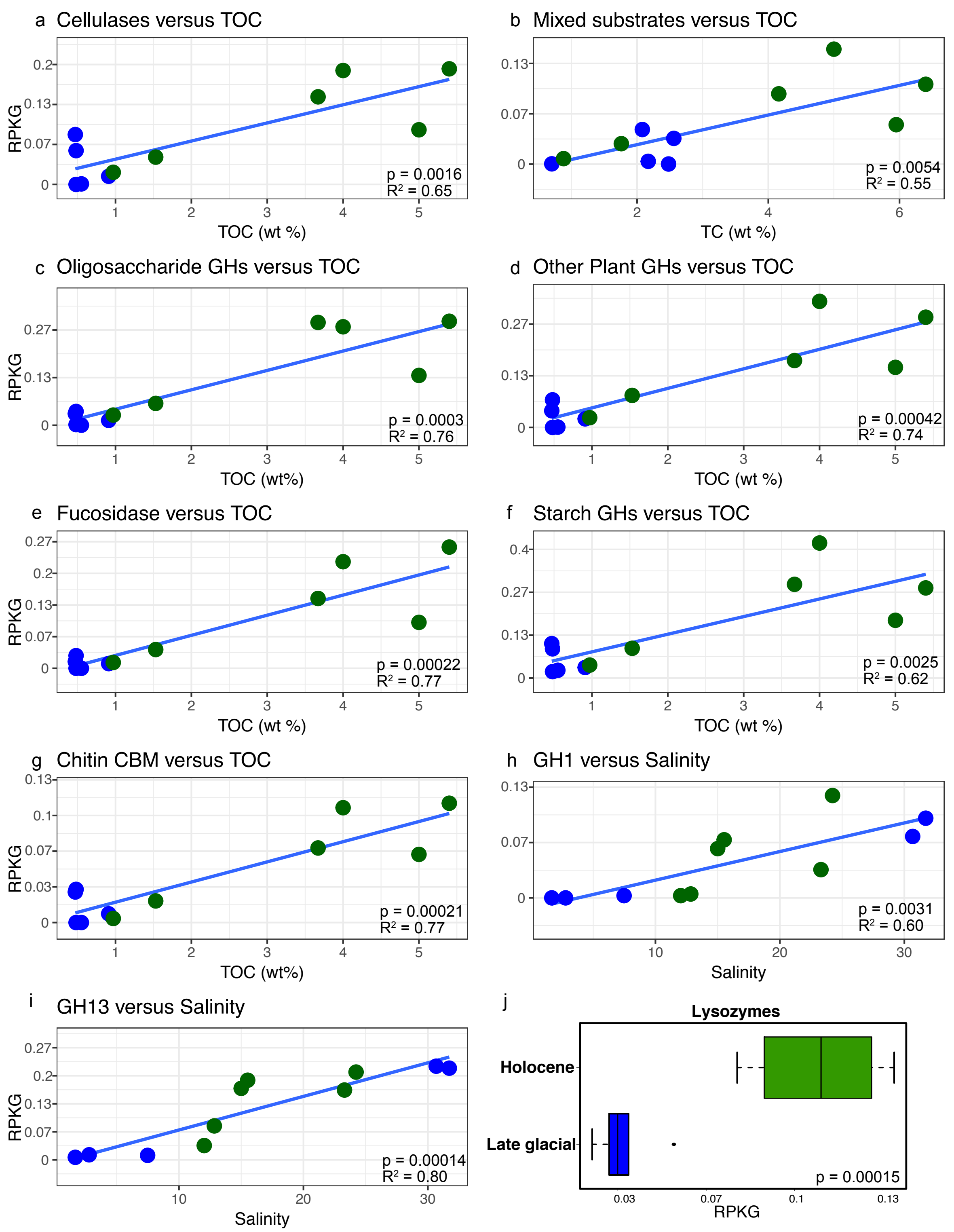

Supplemental Figure 4. Linear models (a-i) or boxplots (j) of abundant ORFs putatively encoding extracellular CAZyme groups compared to select environmental parameters. Blue dots

represent samples deposited during the late Pleistocene and are most glacially influenced, green samples were deposited during the Holocene (i.e. the Ancylus Lake, Littorina Sea, and modern Baltic Sea phases of the Baltic's history). P-values for boxplots are based on t-tests.

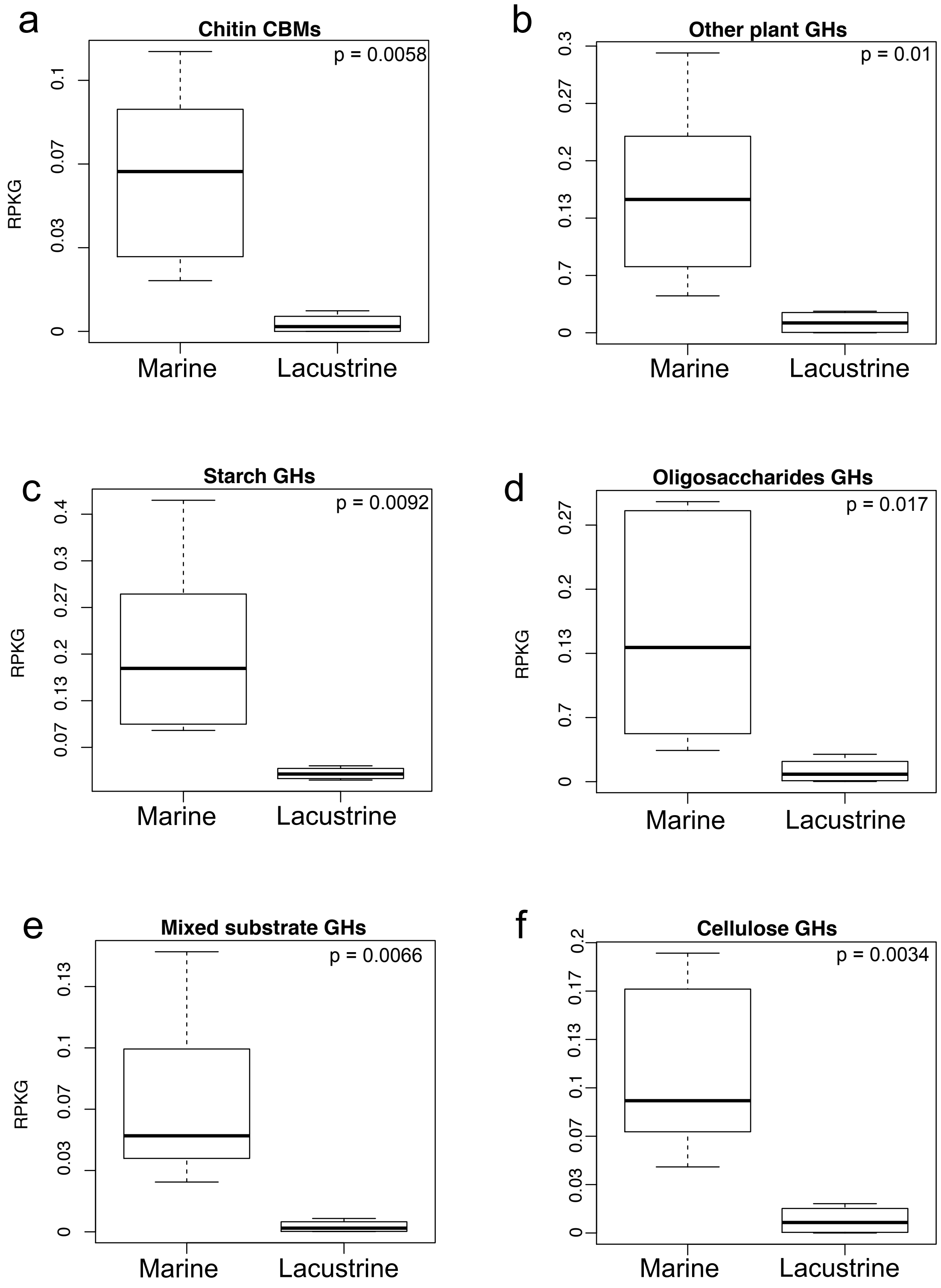
a
Chitin CBMs
p = 0.0058
RPKG
0.1
0.07
0.03
0
Marine
Lacustrine
b
Other plant GHs
p = 0.01
0.3
0.27
0.2
0.13
0.7
0
Marine
Lacustrine
c
Starch GHs
p = 0.0092
RPKG
0.4
0.3
0.27
0.2
0.13
0.07
Marine
Lacustrine
d
Oligosaccharides GHs
p = 0.017
RPKG
0.27
0.2
0.13
0.7
0
Marine
Lacustrine
e
Mixed substrate GHs
p = 0.0066
RPKG
0.13
0.1
0.07
0.03
0
Marine
Lacustrine
f
Cellulose GHs
p = 0.0034
0.2
0.17
0.13
0.1
0.07
0.03
0
Marine
Lacustrine

Supplemental Figure 5. Boxplots showing abundances of ORFs putatively encoding extracellular CAZyme groups by marine versus lacustrine depositional conditions. P-values based on t-tests are shown in the upper right corner of each boxplot.

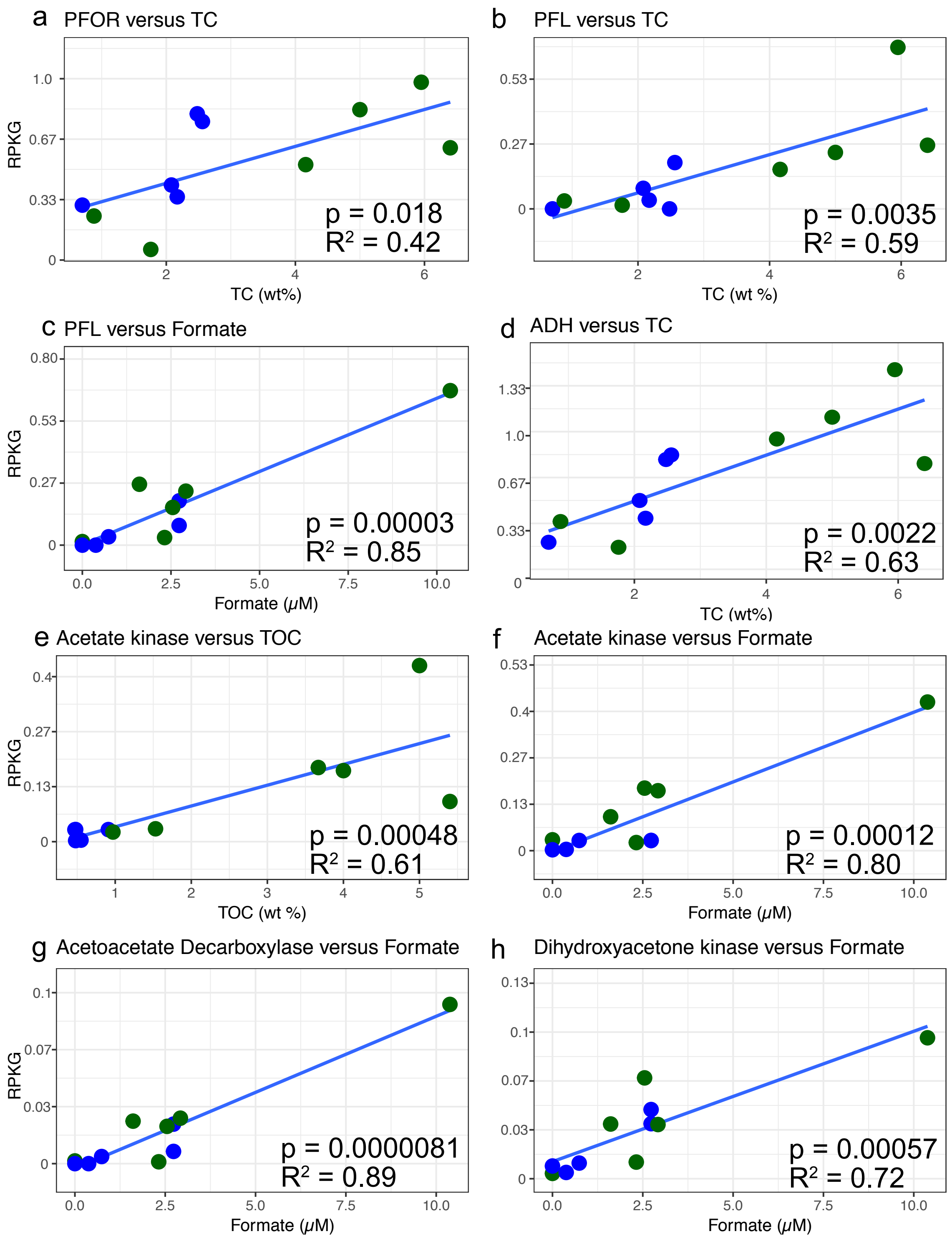

Supplemental Figure 6. Linear models of ORFs putatively encoding fermentation mediating enzymes compared to select environmental parameters. Blue dots represent samples deposited

during the late Pleistocene and are most glacially influenced, green samples were deposited during the Holocene (i.e. the Ancylus Lake, Littorina Sea, and Baltic Sea phases of the Baltic's history).

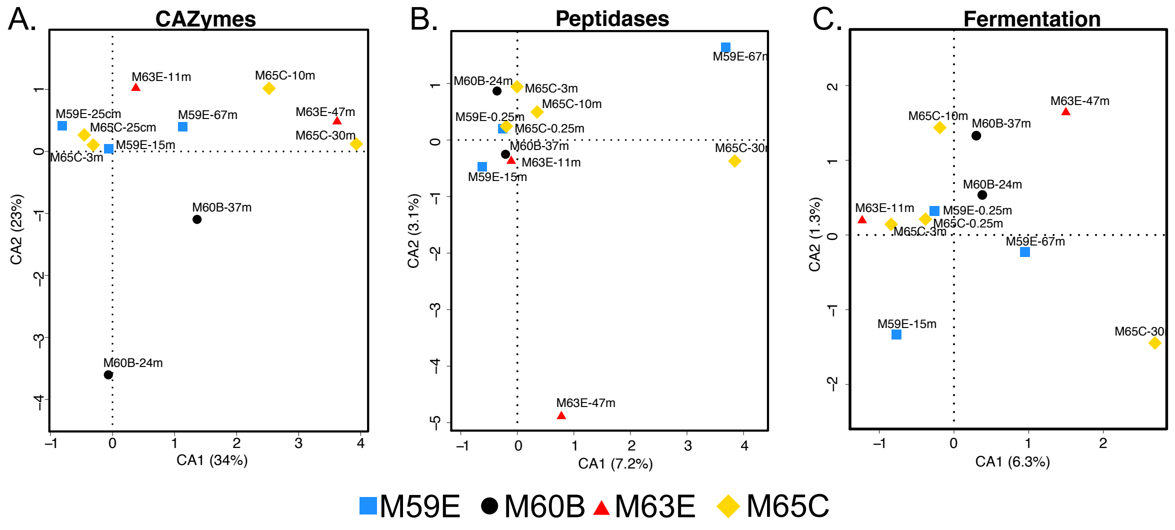

Supplemental Figure 7. Canonical Correspondence Analyses of abundances of ORFs putatively encoding the following groups of enzymes: a) carbohydrate active enzymes; b) peptidases; and c) fermentation mediation proteins.

Supplemental table 1. Sequencing and processing statistics.

	Data Source	Sampling campaign	Site	Sample	Depth (m)	Raw reads	Post-QC reads	Post-dereplication reads	Percent reads mapping to assembly
DNA	This paper	Skaggerak-Kattegat Seabed	M59	SMT	0.25	49,239,081	46,783,878	46,058,076	26.41%
	This paper	IODP x347		5H2	15	29,293,749	28,328,313	26,195,655	35.95%
	Marshall et al.	IODP x347		21H2	67	70,856,845	30,948,382	1,072,012	27.77%
	Marshall et al.	IODP x347	M60	9H2	24	40,486,926	32,499,543	2,018,541	32.63%
	Marshall et al.	IODP x347		13H2	37	54,835,820	41,888,865	4,363,223	32.98%
	Marshall et al.	IODP x347	M63	6H2	12	38,620,470	25,846,898	4,309,404	24.27%
	Marshall et al.	IODP x347		24H2	47	49,069,002	37,775,844	2,211,025	22.53%
	This paper	Bornholm Basin	M65	SMT	0.25	42,069,302	39,698,484	39,156,112	24.96%
	This paper	IODP x347		2H2	3	49,584,551	45,176,765	10,852,368	28.39%
	This paper	IODP x347		4H2	10	138,011,697	127,157,275	17,117,038	15.43%
	Marshall et al.	IODP x347		10H2	30	51,710,778	23,750,244	2,115,020	24.64%
RNA	Zinke et al.	IODP x347	M59	5H2	15	16,968,921	15,232,832	10,908,171	28.36%
	Zinke et al.	IODP x347	M63	6H2	12	17,904,120	16,857,431	12,645,054	24.02%
	Assembly Statistics	**No. of contigs**	**No. of bp in contigs**	**Min bp contigs**	**Max bp contigs**	**Avg bp contigs**	**N50 bp contigs**		
	Co-assembly	557,851	1,072,605,570	1,000	159,114	1,923	1,921		

Supplemental Table 2. PFAM and TIGRFAM identifiers used in this study. Abbreviations are as follows: GH = Glycoside Hydrolase; CBM = Carbohydrate Binding Module; PL = Polysaccharide Lyase.

PFAM or TIGRFAM	Gene name
PF00232	GH1
TIGR03356	GH1 (beta-galactosidase)
PF00703	GH2 (beta-sandwich)
PF02836	GH2 (catalytic domain)
PF00933	GH3 (N-terminal)
PF01915	GH3 (C-terminal)
PF02056	GH4
PF11975	GH4 (C-terminal)
PF00150	GH5
PF14871	GH6
PF00840	GH7
PF01270	GH8
PF00759	GH9
PF00331	GH10
PF02638	GH10-like
PF00457	GH11
PF01670	GH12
PF00128	GH13
PF02922	GH13 (Isoamylase N-terminal domain)
PF14883	GH13 (putative)
PF01373	GH14
PF00723	GH15
PF14885	GH15 (putative)
PF00722	GH16
PF00332	GH17
PF00704	GH18
PF00182	GH19
PF00728	GH20
PF00062	GH22
PF01464	GH23
PF00959	GH24
PF01183	GH25
PF02156	GH26

PF16499	GH27
PF00295	GH28
PF01120	GH29
PF02055	GH30
PF01055	GH31
PF00251	GH32
PF02012	GH33
PF00064	GH64
PF00343	GH35
PF01301	GH35 (catalytic domain)
PF02065	GH36
PF01204	GH37
PF09261	GH38
PF01074	GH38 (N-terminal domain)
PF01229	GH39
PF02449	GH42
PF16126	GH42 (putative)
PF04616	GH43
PF02015	GH45
PF01374	GH46
PF01532	GH47
PF02011	GH48
PF03718	GH49
PF03512	GH52
PF07745	GH53
PF09206	GH54
PF01630	GH56
PF03065	GH57
PF02057	GH61
PF03664	GH62
PF03200	GH63
PF03633	GH65 (C-terminal domain)
PF03632	GH65 (central catalytic domain)
PF03636	GH65 (N-terminal domain)
PF14498	GH65
PF07488	GH67
PF02435	GH68
PF02324	GH70
PF03659	GH71

PF03198	GH72
PF01832	GH73
PF07335	GH75
PF03663	GH76
PF02446	GH77
TIGR00217	GH77 (malQ)
PF05592	GH78
PF03662	GH79
PF03644	GH85
PF07470	GH88
PF05089	GH89
PF07971	GH92
PF10566	GH97
PF08306	GH98
PF16317	GH99
PF03537	GH114
PF12215	GH116
PF04685	GH116 (putative)
PF11308	GH129
PF13204	Cellulase (putative)
PF00734	CBM 1 (fungal cellulose)
PF00553	CBM 2
PF00942	CBM 3 (Cellulose)
PF02018	CBM 4/9
PF02839	CBM 5/12
PF03422	CBM 6
PF02013	CBM 10 (Cellulose)
PF03425	CBM 11
PF00662	CBM 13
PF01607	CBM 14
PF03426	CBM 15
PF03424	CBM 17/28
PF00187	CBM 18
PF03427	CBM 19
PF00686	CBM 20
PF03370	CBM 21
PF03423	CBM 25
PF09212	CBM 27
PF00754	CBM32/47

PF03067	CBM 33
PF02903	CBM 34
PF02973	CBM 40
PF03714	CBM 41
PF05270	CBM 42
PF07983	CBM 43
PF02922	CBM 48
PF09478	CBM 49
PF01476	CBM 50
PF00544	PL1
PF03211	PL3
PF12708	Pectate lyase superfamily
PF06917	Periplasmic pectate lyase
PF14583	Oligogalacturonate lyase
PF14683	PL4, Domain III
PF14686	PL4, Domain II
PF09284	PL4, Domain II
PF05426	PL5
PF02278	PL8
PF05035	2-keto-3-deoxy-galactonokinase
PF09095	Alpha-amylase/4-alpha-glucanotransferase (C-terminal domain)
PF09094	Alpha-amylase/4-alpha-glucanotransferase (central domain)
PF04041	Beta-1,4-mannooligosaccharide phosphorylase
PF08329	Chitinase A
PF06483	Chitinase C
PF00596	Class II Aldolase and Adducin (N-terminal domain)
PF02782	FGGY family of carbohydrate kinases (C-terminal domain)
PF00370	FGGY family of carbohydrate kinases (N-terminal domain)
PF02585	GlcNAc-PI (diacetylchitobiose) deacetylase
PF09985	Glucodextranase (C-terminal binding module, SLH-like)
PF02614	Glucuronate isomerase
PF03328	HpcH/HpaI aldolase/citrate lyase family
PF01081	KDPG and KHG aldolases
PF02610	L-arabinose isomerase
PF11762	L-arabinose isomerase (C-terminal domain)

PF02952	L-fucose isomerase (C-terminal domain)
PF07882	L-fucose isomerase (N-terminal domain II)
PF07881	L-fucose isomerase (N-terminal domain)
PF16863	maltase-glucoamylase (N term)
PF01095	Pectinesterase
PF00294	pfkB family carbohydrate kinase
PF01522	Polysaccharide deacetylase
PF11897	Putative glycogen phosphorylase (DUF3417)
PF05025	RbsD / FucU transport protein
PF01380	Sugar isomerases domain
PF04794	YdjC-like carbohydrate deacetylase
PF00144	Alkaline D-peptidase
PF03576	B-peptidyl aminopeptidase proprotein
PF05343	Broad-specificity exoaminopeptidase
PF03415	Clostripain
PF01364	Peptidase C25 (gingipain)
PF00246	Peptidase M14
PF01546	Peptidase M20 (carboxypeptidase)
PF04389	Peptidase M28
PF01432	Peptidase M3
PF02868	Peptidase M4 (Neutral metallopeptidase; pseudolysin) thermolysin
PF12904	Peptidase M9
PF01435	Peptidase M48
PF02163	Peptidase M50
PF00082	Peptidase S8 (subtilase)
PF10502	Peptidase S26 (signal peptidase)
PF02129	Peptidase S15 (Xaa-Pro dipeptidyl-peptidase)
PF11667	Peptidase Zincin
PF04298	Zinc metallopeptidase II (putative)
PF00871	Aliphatic acid kinase short chain, including acetate kinase and butyrate kinase
PF02550	Acetyl-CoA hydrolase transferase
PF06314	Acetoacetate decarboxylase
PF00465	Alcohol dehydrogenase (Fe containing)
PF00107	Alcohol dehydrogenase (Zinc binding)
PF08240	Alcohol dehydrogenase
PF03306	Acetolactate decarboxylase
PF09163	Formate dehydrogenase

TIGR01583	Formate dehydrogenase
TIGR01591	Formate dehydrogenase
PF06050	2-hydroxylglutaryl-CoA dehydratase
PF02733	Dihydroxyacetone kinase
PF02734	Dihydroxyacetone kinase
PF02288	Diol/glycerol dehydratase
PF02286	Diol/glycerol dehydratase
TIGR01771	Lactate dehydrogenase
PF02901	Pyruvate formate lyase
PF01558	Pyruvate flavoredoxin ferrodoxin oxidoreductase
PF02775	Pyruvate ferrodoxin oxidoreductase
PF00037	Pyruvate ferrodoxin oxidoreductase
PF01855	Pyruvate flavoredoxin ferrodoxin oxidoreductase
PF10371	Pyruvate flavoredoxin ferrodoxin oxidoreductase

Supplemental Table 3. PERMANOVA statistics calculated using abundances of ORFs classified by their annotation, i.e. Glycoside Hydrolases (GHs), Carbohydrate Binding Modules (CBMs), peptidases, and fermentation genes. Statistically significant groups with p-values less than 0.05 are bolded.

Group of genes	Env parameter	P-value	R squared	F statistic
**GHs**	**Sediment age**	**0.043**	**0.245**	**2.915**
**GHs**	**Glacial**	**0.035**	**0.22682**	**2.6402**
**GHs**	**Marine vs. lacustrine**	**0.003**	**0.44**	**7**
**GHs**	**Salinity**	**0.01**	**0.34**	**4.6**
**GHs**	**TC**	**0.005**	**0.34**	**4.7**
**GHs**	**TOC**	**0.009**	**0.32**	**4.2**
GHs	Formate	0.177	0.164	1.771
GHs	Acetate	0.352	0.366	1.03
GHs	Propionate	0.217	0.14	1.412
GHs	Butyrate	0.143	0.163	1.748
CBMs	Sediment age	0.051	0.256	3.101
CBMs	Glacial	0.092	0.20283	2.29
**CBMs**	**Marine vs. lacustrine**	**0.008**	**0.3747**	**5.3931**
**CBMs**	**Salinity**	**0.036**	**0.284**	**3.574**
**CBMs**	**TC**	**0.003**	**0.46181**	**7.7228**
**CBMs**	**TOC**	**0.005**	**0.42868**	**6.753**
CBMs	Formate	0.072	0.261	3.175
CBMs	Acetate	0.221	0.144	1.51
CBMs	Propionate	0.263	0.85	0.086
CBMs	Butyrate	0.535	0.078	0.76
Peptidases	Age	0.199	0.134	1.395
Peptidases	Glacial	0.284	0.11596	1.1805
**Peptidases**	**Marine vs. lacustrine**	**0.032**	**0.3624**	**5.1155**
**Peptidases**	**Salinity**	**0.038**	**0.29756**	**3.8125**
**Peptidases**	**TC**	**0.002**	**0.42888**	**6.7584**
**Peptidases**	**TOC**	**0.023**	**0.32729**	**4.3787**
**Peptidases**	**Formate**	**0.021**	**0.261**	**1.181**
Peptidases	Acetate	0.099	0.221	2.55
Peptidases	Propionate	0.247	0.136	1.412
Peptidases	Butyrate	0.263	0.14	1.416

**Fermentation**	**TOC**	**0.021**	**0.36374**	**5.1452**
**Fermentation**	**TC**	**0.002**	**0.53839**	**10.497**
Fermentation	Glacial (yes or no)	0.284	0.116	1.213
Fermentation	Marine vs. lacustrine	0.088	0.2415	2.865
Fermentation	Salinity	0.151	0.211	2.408
Fermentation	Approx Age	0.232	0.14	1.4653
Fermentation	Formate	0.051	0.32	4.231
Fermentation	Acetate	0.093	0.228	2.654
Fermentation	Propionate	0.514	0.072	0.694
Fermentation	Butyrate	0.802	0.033	0.699
